# Notes

**Published:** 1898-09

**Authors:** 


### Notes.

### A REMARKABLE MAN.

Without doubt, Dr. N. Senn is a remarkable man. Despite the fact of his being Lieutenant Colonel, U. S. V., chief of the operating staff with the army in the field, he manages to snatch time here and there sufficient to keep up a discharge of literary pellets which find lodgment in the leading medical weeklies of the country. No rapid-fire gun ever delivered itself more incessantly than the chief of the operating staff with the army in the field.

Could these epistolary discharges be collected into one, they would make a volume equal in size to a patent office report—and probably possess the same thrilling interest.

---

One of the new (and at the same time "ol") Germansynthetic compounds is appropriately called Orphol. But unfortunately the name does not refer to the number already on the market.

Attention was called some time ago to *anusol* as a remedy for piles. It would seem that some of the *Fliegende Blatter* staff had a hand in christening these compounds.

---

Dr. James A. Ellis, formerly of Richmond, Va., has made his home in Atlanta.

---

Dr. Floyd W. McRae has been appointed to deliver the address on surgery at the next meeting of the American Medical Association.

---

### FOR SALE.

A Ranney Cabinet Battery made by Waite & Bartlett. Forty cells; height, 69 inches; length, 41 inches; depth, 22 inches. Good condition and cost when new $260. Address Box 436, Atlanta, Ga.

